# ADHD medication dispensing trends in Dutch youth before and after the implementation of the Youth Act (2010–2022)

**DOI:** 10.1007/s00787-025-02791-w

**Published:** 2025-06-23

**Authors:** Lisa T. Ringeling, Aarushi Srivastava, Ravish N. Gangapersad, Jens H. J. Bos, Brenda C. M. de Winter, Eelko Hak, Manon H. J. Hillegers, Birgit C. P. Koch, Catharina C. M. Schuiling-Veninga, Bram Dierckx

**Affiliations:** 1 Department of Hospital Pharmacy, Erasmus University Medical Center, Rotterdam, The Netherlands; 2 Department of Child and Adolescent Psychiatry/Psychology, Erasmus University Medical Center, Rotterdam, The Netherlands; 3 Erasmus School of Economics, Erasmus University Rotterdam, Rotterdam, The Netherlands; 4 Department of Pharmacotherapy, -Epidemiology and -Economics (PTEE), University of Groningen, Groningen, the Netherlands; 5 Ringeling at Erasmus MC, University Medical Center Rotterdam, Wytemaweg 80, PO box 2060, Rotterdam, 3000 CB The Netherlands

**Keywords:** Youth Act, ADHD medication, Psychostimulants, Dutch youth, Prevalence, Incidence, Dosages, Duration of use

## Abstract

Objective: The Youth Act, implemented by the Dutch government in 2015, aimed to provide more efficient, coherent, and cost-effective care tailored to the specific needs of children, while also reducing medicalization. Between 2003 and 2013, there was a four-fold increase in methylphenidate prescriptions among Dutch children aged 4 to 18 years. However, the impact of the Youth Act on the ADHD medication dispensing remains unassessed. This study investigated changes in ADHD medication dispensing patterns among Dutch youth following the implementation of the Youth Act. Method: A before-after retrospective cohort study was conducted using data from the Dutch community pharmacy-based IADB.nl database among 137,684 youths aged 0–19 years using ADHD medication between 2010 and 2022. Results: A significant decrease in overall ADHD medication dispensing prevalence rates per thousand youths was observed in 2022 compared to 2015 (35.2 vs. 45.7, *p* < 0.001). Methylphenidate accounted for approximately 87% of all ADHD medication dispensing. Incidence rates decreased significantly among children aged 7–12 from 2015 to 2022 (9 vs. 7.3, *p* < 0.001), while incidence rates increased significantly in adolescent youths (4 vs. 4.8, *p* = 0.008), particularly among adolescent females (3.5 vs. 5.3, *p* < 0.001). Females had lower prevalence rates and shorter durations of ADHD medication use compared to males, with no difference in daily dose observed. Conclusion: ADHD medication dispensing among Dutch youth declined following the 2015 implementation of the Youth Act. However, other factors such as the DSM-5 revision, COVID-19, and medication shortages likely contributed to this decrease, suggesting a multifactorial explanation for the observed changes in ADHD medication dispensing.

**Supplementary Information:**

The online version contains supplementary material available at 10.1007/s00787-025-02791-w.

## Introduction

Attention Deficit Hyperactivity Disorder (ADHD) is a common psychiatric disorder among children and adolescents. It is characterized by developmentally inappropriate levels of inattention, impulsivity, and hyperactivity, as defined by the Diagnostic and Statistical Manual of Mental Disorders, 5th edition (DSM-5) [1]. The treatment for ADHD depends on the age, developmental stage, and the severity of symptoms, and may involve non-pharmacological interventions, pharmacological therapy, or a combination of both [2]. In addition, a substantial proportion of children with ADHD (approximately 60–100%) exhibit comorbidities such as autism spectrum disorder, psychotic disorders, anxiety, and depression [3]. The treatment of these comorbidities often requires the use of antipsychotics or antidepressants, depending on the burden of disease [4]. Considering that ADHD in children is often connected to comorbidities and co-medication, this suggests the presence of polypharmacy in this specific population.

While medication plays a crucial role in the treatment of ADHD, the Dutch government has prioritized efforts to reduce reliance on medication in children and adolescents as part of the Youth Act implemented in 2015. The Youth Act was established to decentralize youth care services from the national government to the municipalities. This transfer of responsibilities aimed to provide more efficient, coherent, and cost-effective care tailored to the specific needs of children and their families, enabling early detection and support for social or behavioral problems. Additionally, part of the Youth Act was to de-medicalize Dutch youth in mental healthcare. De-medicalization refers to reducing dependence on psychotropic medication for addressing deviant behavior in children and adolescents, advocating instead for alternative approaches such as social support and behavioral interventions [5, 6]. Despite the effectiveness of ADHD medication, potential side effects and safety concerns have predominantly been studied during short-term use. Thus, little is known about the safety after long-term use of ADHD medication [7].

In 2012, psychoactive medications were prescribed to 200,000 (5%) of nearly 4 million youths aged 0 to 21 years, with methylphenidate being by far the most commonly used psychoactive agent. Between 2003 and 2013, there was a four-fold increase in the number of prescriptions of methylphenidate among Dutch children aged 4 to 18 years [8]. Before the implementation of the Youth Act, the prevalence of ADHD among Dutch youth ranged from 0.5 to 24%, partly due to the absence of an objective measurement method to assess the degree of hyperactivity, impulsivity, and/or inattention [9]. Globally, the average prevalence of ADHD is between 2 and 7% with an average of 5.3%, with boys experiencing two to three times higher prevalence rates than girls [10]. The period after the implementation of the Youth Act holds particular significance as concerns were raised regarding the municipalities’ capacity to effectively organize mental youth care and accurately identify cases that require specialized treatment, including medical treatment. Consequently, access to suitable mental health care services may have been affected [5]. While ADHD medication use in Dutch youth is observed extensively before 2015 [9], the period following the implementation of the Youth Act remains understudied. Despite data from the Dutch Institute for Responsible Medication Use indicating a slight decrease in the number of ADHD medication users aged 2 to 17 years in 2020 compared to 2019 [11], no studies have been conducted after the implementation of the Youth Act to observe its effects on the use of ADHD medication in Dutch youth. This raises the question: has the use of ADHD medication in Dutch youth changed following the implementation of the Youth Act? Considering that, among other objectives, the Youth Act aims to minimize prescriptions and de-medicalize mental healthcare, it is hypothesized that the Youth Act has led to a decrease in ADHD medication use in Dutch youth. We investigated this hypothesis by analyzing the dispensing of ADHD medication among youth in the Netherlands before (2010–2015) and after (2016–2022) the implementation of the Youth Act.

## Method

### Data source

We conducted a retrospective before-after cohort study to evaluate the impact of the Youth Act implementation on ADHD medication utilization. The data needed for this research were collected from the IADB.nl pharmacy prescription database of the University of Groningen [12]. Currently, the database includes data from 125 community pharmacies in the Northern and Eastern parts of the Netherlands. These pharmacies cover data of approximately 1,300,000 patients, which are representable for the general Dutch population [13]. The IADB.nl database collects patient data from the participating community pharmacies with an opt-out approach. Approval of the medical ethics committee was not required since the data and records in the IADB.nl database are anonymized. Additionally, data collection was in accordance with the national and European privacy guidelines regarding the handling of human data.

### Study sample

Patients aged 0 to 19 years who received ADHD medications between January 1, 2010, and December 31, 2022, were included. Data included general information (age, sex, appearance dates in the database, yearly population estimates) and dispensing details (dispensing date, units delivered, daily dose, DDD). ADHD medication classes were defined using the World Health Organization’s Anatomical Therapeutic Chemical/Defined Daily Dose Classification System, with specified ATC codes for psychostimulants (N06B), and centrally acting hypertensives (C02AC). Co-medication data included ATC codes for antidepressants (N06A), psycholeptics and psychoanaleptics in combination (N06C), antipsychotics (N05A), anxiolytics (N05B), and hypnotics and sedatives (N05C) [14].

### Data analysis

#### Prevalence and incidence

Prevalence and incidence rates of ADHD medication dispensing were calculated per year from 2010 to 2022. The incidence and prevalence rates for 2010, 2015 and 2022 were compared and statistically analyzed to observe differences between the first observed year (before implementation), the year of implementation and the last observed year (after implementation). The rates were further stratified by sex and age groups (0–6 years, 7–12 years, and 13–19 years). The ages in the database were based on the first of January of the year of the dispensing. A new user was defined as someone who had been present in the database for at least 90 days before their first drug dispensing. To calculate prevalence and incidence rates, the number of users was divided by the total underlying population present in the IADB.nl database and presented per thousand. The total underlying population is an annual estimate and can be viewed as a dynamic stationary cohort without substantial changes in size throughout the year, ranging from 178,270 to 275,861 individuals aged 0–19 years between 2010 and 2022.

#### Dose analysis

Dose analysis was performed for the psychostimulant drugs, clonidine and guanfacine. Mean daily dosages per year were analyzed using the defined daily dose (DDD) [14]. The mean daily doses are presented as the value ± standard deviation (SD).

#### Duration of use

The duration of use for ADHD medication was calculated for incident users in months using median survival times determined by the Kaplan Meier estimator. The start of an episode of ADHD medication use was defined as the date of dispensing. An episode ended when the duration for which the medication was dispensed plus an additional 90 days had elapsed, and the youth could still be followed in the database. All other cases were classified as censored. The median survival times were calculated for sex, age groups (at the time of ADHD medication initiation), and specific ADHD medications.

#### Concurrent psychotropic medication

Concurrent psychotropic treatment was defined as a psychotropic dispensation being issued within the start and stop dates of an ADHD medication dispensation.

#### Statistical analysis

Statistical analysis was performed using SPSS for Windows, version 28.0.1.0 (142), and Microsoft Excel 2016. Differences were considered statistically significant at *p* < 0.05. Chi-square tests were used to compare proportions.

## Results

Within the IADB.nl database, a total of 137,684 unique individuals aged 0–19 years were dispensed ADHD medications at least once between 2010 and 2022. The cohort was stratified into 2,637 individuals aged 0–6 years, 50,175 individuals aged 7–12 years, and 84,872 individuals aged 13–19 years, with 94,790 males and 42,894 females.

### Prevalence

**Fig. 1:**
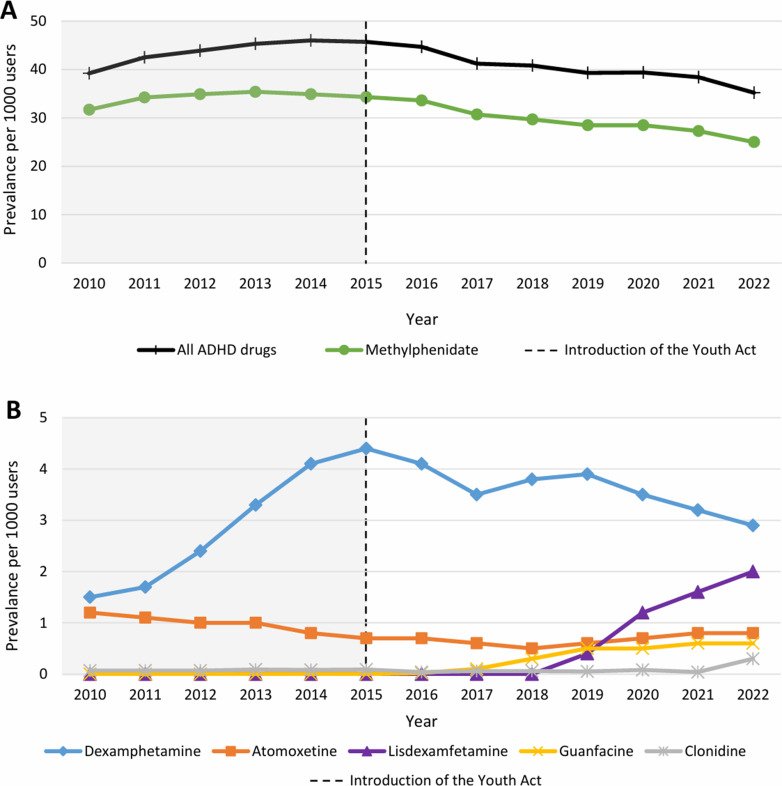
Prevalence per thousand youths of the six most commonly dispensed ADHD medications in 0–19-year-olds. (**A**) The prevalence of all six ADHD medications combined and of methylphenidate dispensing. (**B**) The prevalence of dexamphetamine, atomoxetine, lisdexamfetamine, guanfacine and clonidine dispensing

The yearly total prevalence rate of ADHD medication dispensing significantly increased from 39.2 per thousand youths in 2010 to 45.7 per thousand youths in 2015 (*p* < 0.001). However, after the implementation of the Youth Act in 2015, the prevalence rate demonstrated a steady decline, reaching its lowest point by 2022, at 35.2 per thousand youths (*p* < 0.001 compared to both 2010 and 2015) (Fig. 1A). The prevalence rates stratified by age, sex, and year are presented in Table 1. The lowest yearly prevalence rates were observed for 0–6-year-olds across all years and sexes. The highest prevalence rates were observed in males aged 7–12 and 13–19 years. Additionally, females had consistently lower prevalence rates across all age groups and years compared to males.

**Table 1:** Prevalence (per thousand youths) of ADHD medication dispensing among Dutch youth aged up to 19 years

	2010^A^	2015^B^	2022^C^	*p*-value		
*Total*				2010 vs. 2015	2015 vs. 2022	2010 vs. 2022
All age groups	39.2	45.7	35.2	< 0.001 ↑	< 0.001 ↓	< 0.001 ↓
0–6 years	4.4	2.3	1.3	< 0.001 ↓	< 0.001 ↓	< 0.001 ↓
7–12 years	59.4	57.2	37.9	0.108	< 0.001 ↓	< 0.001 ↓
13–19 years	54.5	73.4	60.2	< 0.001 ↑	< 0.001 ↓	< 0.001 ↓
*Males*						
0–6 years	7.0	3.5	1.9	< 0.001 ↓	< 0.001 ↓	< 0.001 ↓
7–12 years	90.6	84.3	56.2	0.004 ↓	< 0.001 ↓	< 0.001 ↓
13–19 years	75.5	96.4	69.0	< 0.001 ↑	< 0.001 ↓	< 0.001 ↓
*Females*						
0–6 years	1.6	1.0	0.7	0.030 ↓	0.090	< 0.001 ↓
7–12 years	26.8	28.7	18.9	0.158	< 0.001 ↓	< 0.001 ↓
13–19 years	35.0	50.6	51.2	< 0.001 ↑	0.685	< 0.001 ↓

^A^*n* = 90,171 males, *n* = 88,099 females, *n* = 178,270 total

^B^*n* = 138,213 males, *n* = 133,729 females, *n* = 271,942 total

^C^*n* = 122,087 males, *n* = 116,645 females, *n* = 238,732 total

↑ = significant increase

↓ = significant decrease

The most frequently dispensed ADHD medication in the database was methylphenidate (86.6%), followed by dexamphetamine (9.0%), atomoxetine (2.3%), lisdexamfetamine (1.2%), guanfacine (0.6%), and clonidine (0.2%). Following the implementation of the Youth Act, there was a notable shift in medication trends. While the overall prevalence of ADHD medication dispensing decreased, there was an increase in the use of the less frequently dispensed medications (Fig. 1). Methylphenidate, which accounted for approximately 87% of all dispensed ADHD drugs, demonstrated prevalence rates ranging from 31.7 per thousand youths in 2010, peaking at 35.4 per thousand youths in 2013, and declining to 25 per thousand youths by 2022. The prevalence of dexamphetamine also decreased considerably following the implementation of the Youth Act, dropping from 4.4 per thousand youths in 2015 to 3.5 per thousand youths in 2017, and further to 2.9 per thousand youths in 2022. In contrast, lisdexamfetamine showed a notable increase in prevalence with no dispensations recorded before 2019, and rates reaching 2 per thousand youths by 2022.

### Incidence

**Fig. 2:**
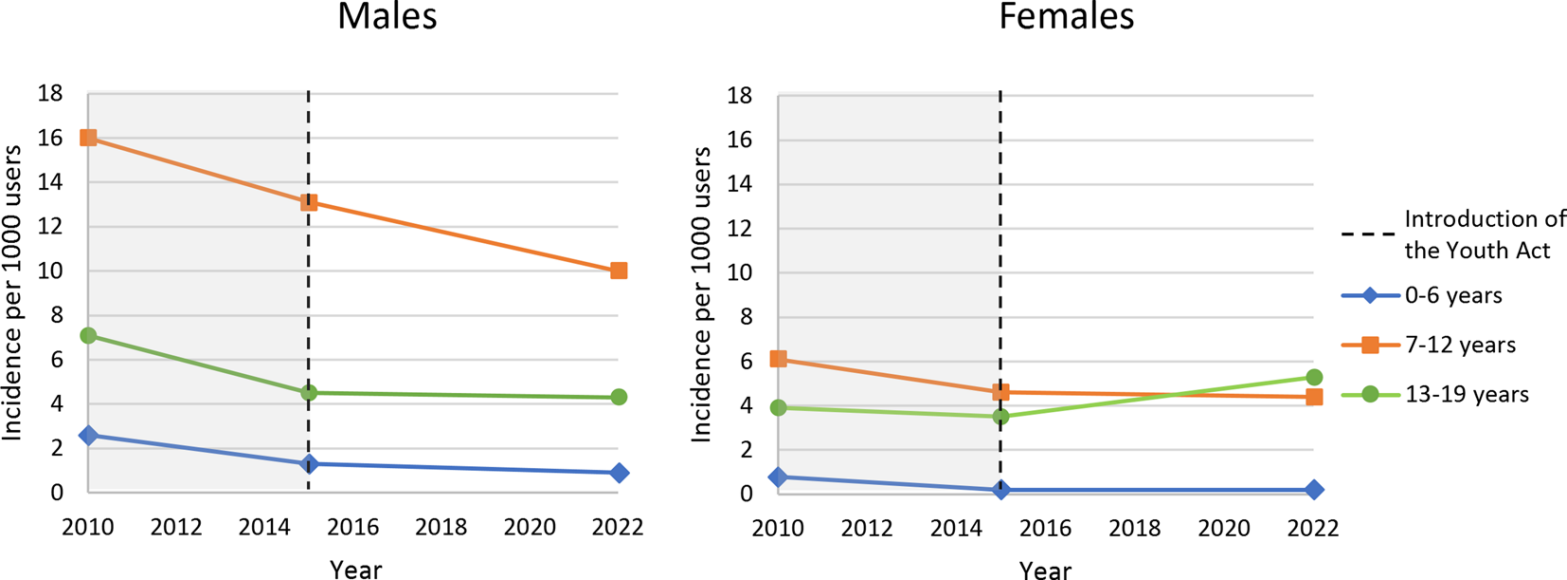
Incidence per thousand users of ADHD medication among males and females per age group

The overall incidence rates varied between 5.9 per thousand in 2010 and 4.1 per thousand in 2022, reaching the lowest point in 2016 with 3.6 per thousand youths. However, the decrease in overall incidence rates of ADHD medication use between 2015 and 2022 was not significant (*p* = 0.127). The incidence rates, stratified by age, sex, and start year, are presented in Table 2. For almost all age groups and in both males and females, the incidence rates decreased significantly when comparing 2010 with 2022. However, after 2015, the incidence rates for females aged 13–19 years increased significantly, rising from 3.5 per thousand females in 2015 to 5.3 per thousand females by 2022 *(p <* 0.001*)*. Males had an overall higher incidence rate compared to females, especially in the age category of 7–12-year-olds (Fig. 2).

**Table 2:** Incidence (per thousand youths) of ADHD medication dispensations among Dutch youth aged up to 19 years

	2010^A^	2015^B^	2022^C^	*p*-value		
*Total*				2010 vs. 2015	2015 vs. 2022	2010 vs. 2022
All age groups	5.9	4.4	4.1	< 0.001 ↓	0.127	< 0.001 ↓
0–6 years	1.7	0.8	0.6	< 0.001 ↓	0.093	< 0.001 ↓
7–12 years	11.1	9.0	7.3	< 0.001 ↓	< 0.001 ↓	< 0.001 ↓
13–19 years	5.5	4.0	4.8	< 0.001 ↓	0.008 ↑	0.041 ↓
*Males*						
0–6 years	2.6	1.3	0.9	< 0.001 ↓	0.121	< 0.001 ↓
7–12 years	16	13.1	10	0.002 ↓	< 0.001 ↓	< 0.001 ↓
13–19 years	7.1	4.5	4.3	< 0.001 ↓	0.573	< 0.001 ↓
*Females*						
0–6 years	0.8	0.2	0.2	< 0.001 ↓	0.488	< 0.001 ↓
7–12 years	6.1	4.6	4.4	0.011 ↓	0.653	0.004 ↓
13–19 years	3.9	3.5	5.3	0.295	< 0.001 ↑	0.006 ↑

^A^*n* = 90,171 males, *n* = 88,099 females, *n* = 178,270 total

^B^*n* = 138,213 males, *n* = 133,729 females, *n* = 271,942 total

^C^*n* = 122,087 males, *n* = 116,645 females, *n* = 238,732 total

↑ = significant increase

↓ = significant decrease

### Dose analysis

For the six dispensed ADHD medications, the database contained a total of 113,326 dispensations. From these dispensing data, mean doses for all ADHD medications were obtained in milligrams and categorized by year, age group, and sex (see appendix I). Mean daily doses remained similar for all ADHD medications between 2010 and 2022. The mean daily dose of the most dispensed drug, methylphenidate, for 0–6-year-olds was 12 mg and was similar for both males and females during all years. For 7–12-year-olds, the mean daily dose of methylphenidate increased to 21 mg for males and females across all years. Finally, for 13–19-year-olds, the mean daily dose of methylphenidate was 33 mg, with no significant differences in daily doses between sexes observed across all years. The remaining ADHD medications also did not show significant changes in mean daily dose during the research period.

### Duration of use

**Fig. 3:**
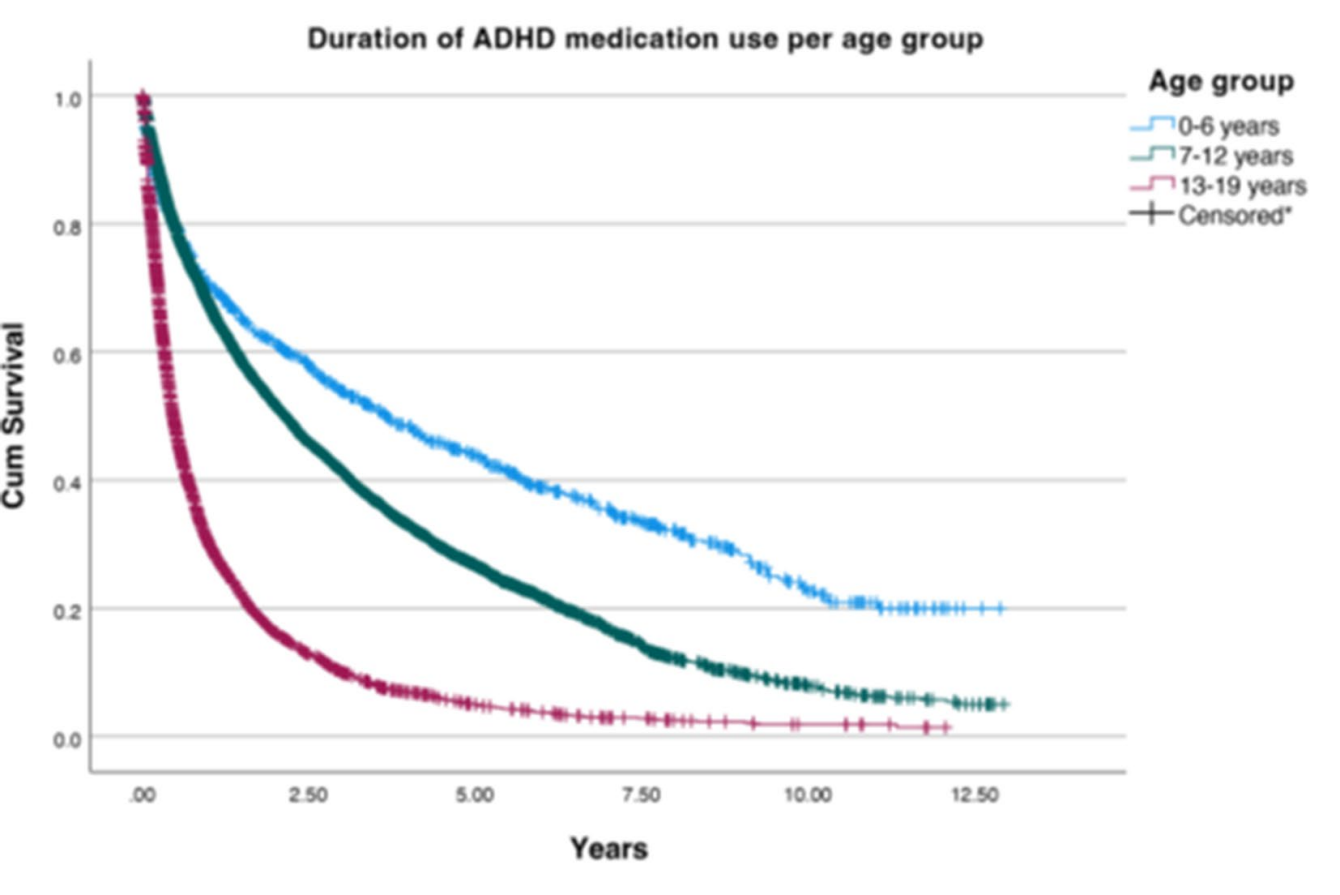
Duration of ADHD medication use per age group. *Each vertical line represents an individual that has stopped using ADHD medication

**Fig. 4:**
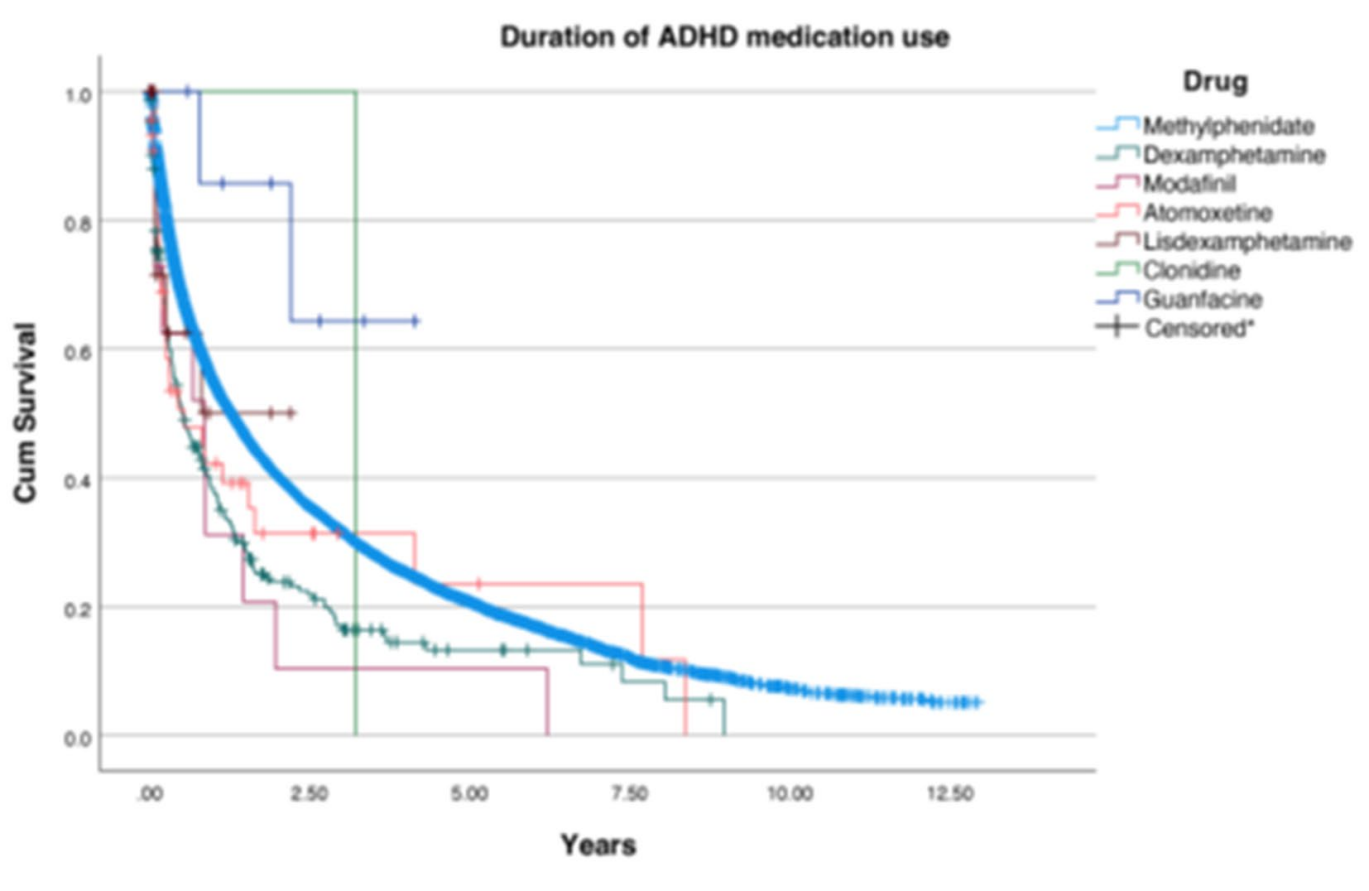
Duration of each ADHD medication. *Each vertical line represents an individual that has stopped using ADHD medication

The duration of ADHD medication use was analyzed by both age category and by specific drug in starters, as illustrated in Figs. 3 and 4, respectively. When looking at the overall median duration of use for all the age groups, it was observed that 0–6-year-olds had the longest median duration of use, at 5.3 years, compared to a median duration of use of 3.5 years for 7–12-year-olds and 1.2 years for 13–19-year-olds. The 7–12-year-old children demonstrated 5,056 censored data points, suggesting a higher rate of treatment discontinuation compared to 525 for 0–6-year-olds and 3,998 for 13–19-year-olds. Most of the 13–19-year-olds discontinued their medication within 2.5 years of the treatment, while the younger age groups persisted in ADHD medication use up to approximately 12.5 years. Both sexes included patients with ADHD medication use up to 12.5 years. However, girls had a shorter median duration of use at 2.2 years, compared to 3.2 years for boys (see appendix II). Based on the median duration of use specified by drug, it can be concluded that most youths in the IADB.nl database used methylphenidate the longest (150 months), followed by dexamphetamine (approximately 110 months) and atomoxetine (100 months).

### Concurrent psychotropic medication

Selective serotonin reuptake inhibitors (SSRIs), benzodiazepines, and tricyclic antidepressants (TCAs) were the most frequently dispensed concurrent medications each year. Of the 20,982 youths with concomitant medication, 2,635 (12.5%) used an SSRI, 1,421 (6.8%) used a benzodiazepine, and 1,165 (5.6%) used a TCA.

## Discussion

Our analysis revealed a notable trend in ADHD dispensing rates following the implementation of the Youth Act in 2015. Initially, prevalence rates increased significantly from 39.2 per thousand youths in 2010 to a peak of 45.7 per thousand youths in 2015. However, after 2015, there was a steady decline in prevalence rates, reaching the lowest number of users by 2022 at 35.2 per thousand youths. Additionally, we found that males were dispensed ADHD medication more frequently than females, with notably higher prevalence rates, particularly in the age groups of 7–12 years and 13–19 years. However, males in these age groups also experienced the most significant decline in prevalence rates after 2015. Furthermore, males had longer durations of treatment compared to females. In contrast, females aged 13–19 years showed a significant increase in incidence rates, rising from 3.5 per thousand in 2015 to 5.3 per thousand by 2022. Among the various ADHD medications, methylphenidate was the most dispensed drug (86.6%) during the study period, followed by dexamphetamine (9.0%), atomoxetine (2.3%), and lisdexamfetamine (1.2%). The prevalence rates of all ADHD medications declined after the implementation of the Youth Act, except for lisdexamfetamine where an increase in prevalence rates was observed after 2018.

Consistent with earlier findings in adults between 2004 and 2014 [15], methylphenidate was the most dispensed ADHD drug in our findings. Another study illustrated an increase in methylphenidate use in youths from 2003 until 2013, followed by a stabilization in subsequent periods [16]. Our data on youths aligns with this trend, showing an increase from 31.7 per thousand youths in 2010 to the highest prevalence rate of 35.4 per thousand youths in 2013. The following years up to 2022 demonstrated a sustained decrease, reaching the lowest prevalence of 25.0 per thousand youths in 2022. Moreover, the Dutch Foundation for Pharmaceutical Key Figures (Stichting Farmaceutische Kerngetallen) reported a decrease in methylphenidate usage among individuals under 18 years old between 2017 and 2021 [17]. This reduction was observed across all age groups and both sexes in our findings, indicating a declining trend in the prevalence of ADHD medication dispensing after the implementation of the Youth Act in 2015. Besides the decline in methylphenidate dispensing after 2015, our findings indicate a noticeable shift towards other ADHD medications. The decrease in the methylphenidate dispensing prevalence and the concurrent increase in the prevalence rates of dexamphetamine, lisdexamfetamine, and atomoxetine dispensing suggest that patients may be switching between these medications. Additionally, the prevalence of dexamphetamine dispensing decreased from 4.4 per thousand youths in 2015 to 3.5 per thousand youths in 2017, followed by a brief increase and subsequent decrease to 3.2 per thousand youths in 2020, which may be associated with the increasing prevalence of lisdexamfetamine dispensing. Lisdexamfetamine was officially registered in 2018, marking the beginning of its gradual increase in dispensing, which peaked at a prevalence of 2 per thousand youths in 2022. The rapid rise suggests that individuals may have switched from dexamphetamine to lisdexamfetamine due to its beneficial pharmacokinetic profile, which leads to a longer duration of activity [18]. The dispensing data indicate that youths initially received methylphenidate, followed by dexamphetamine, and subsequently lisdexamfetamine, atomoxetine, or guanfacine. This aligns with the steps described in the treatment approach according to the Dutch guidelines [2]. It is important to recognize that these guidelines are used as guidance for prescribers, and treatments are optimized and personalized for each patient. This variability in treatment approach could also explain the absence of a drastic decrease in overall medication use among youth (39.2 per thousand youths in 2010, compared to 35.2 per thousand youths in 2022), as the severity of ADHD symptoms among individuals in the study population may vary significantly.

Besides the implementation of the Youth Act, there have been other factors between 2010 and 2022 that could have played a role in the use of ADHD medication among youths. Firstly, lower prevalence rates among children aged 0–6 years align with the current Dutch treatment guidelines, as ADHD medications are not approved for children under 6 years [2]. In some cases, however, a specialist may decide to prescribe off-label. Nevertheless, our results indicate a significant decrease in dispensations from 4.4 per thousand youths in 2010 to 1.3 per thousand youths in 2022 (*p* < 0.001) for children aged 0 to 6 years. Secondly, the DSM-5 was published in 2013, with subtle yet significant changes to diagnostic criteria and treatment approaches for ADHD compared to the DSM-IV [19]. Notably, in the DSM-5, the age range broadened for symptom onset to before 12, potentially leading to more children and adolescents meeting diagnostic criteria for ADHD. Additionally, the symptom threshold was reduced from at least six symptoms to five for adolescents and adults, recognizing that symptoms may decrease with age. Furthermore, the DSM-5 emphasized the assessment and diagnosis of comorbid conditions alongside ADHD, such as anxiety, depression, and learning disabilities, which may lead to increased polypharmacy in treatment regimens. Our study observed the use of concurrent medication among youths receiving ADHD medications, which aligned with existing literature on co-medication use in children with ADHD [3]. Our findings revealed that 18.1% of participants were dispensed antidepressants, such as SSRIs or TCAs, alongside ADHD medication, and 6.8% were dispensed anxiolytic benzodiazepines. The dispensations of these co-medications underscore the prevalence of co-morbid mental health disorders, such as depression and anxiety, among children and adolescents with ADHD [4]. Thirdly, the potential impact of the coronavirus disease 2019 (COVID-19) pandemic and subsequent school closures and home schooling on ADHD medication dispensing patterns remains unclear. Gimbach et al. observed a rise in ADHD prescriptions across Europe, including the Netherlands [20]. However, our data—focused specifically on Dutch youth aged 0–19—show a decline in dispensing between 2019 and 2022. This contrast may reflect age-specific responses to the pandemic. Among adults, increased mental health awareness and routine disruptions may have contributed to more diagnoses and treatment. In children, by contrast, school closures and reduced access to diagnostic services likely resulted in fewer new cases and temporary discontinuation of medication during remote learning. Additionally, structural factors such as the Youth Act may have created barriers to care for youth, including longer waiting times and administrative hurdles. Gangapersad et al. reported a moderate increase in antipsychotic medication use among youth up to 19 years old during the COVID-19 pandemic [21], suggesting broader changes in pediatric psychotropic dispensing. Additional research is required to clarify the specific effects of COVID-19 on ADHD medication usage in Dutch youth.

Lastly, medication shortages have posed a substantial problem in the Netherlands during the last two years of our study period. According to the Royal Dutch Society for the Promotion of Pharmacy (KNMP), there were 1,514 drug shortages in 2022, including key ADHD medications such as methylphenidate, lisdexamfetamine, and atomoxetine [22]. These shortages may have influenced dispensing trends in 2021 and 2022. KNMP Farmanco, the national medication shortage monitoring system, provided only general timeframes of medication unavailability but lacked detailed data on stock levels, shortage severity, causes, and substitution practices. Although switching to alternative brands was often possible, delays or reluctance to switch may have affected medication use. Due to these data limitations, we were unable to quantify the exact impact of the shortages.

Regarding differences between sexes, our findings showed a significant increase in ADHD medications incidence rates among female adolescents (3.5 per thousand females in 2015 vs. 5.3 per thousand in 2022, *p* < 0.001). This development aligns with literature highlighting the growing awareness of ADHD among marginalized populations, such as females and older individuals [23, 24]. Females often receive an ADHD diagnosis later than males, likely due to differences in symptom manifestation. Males with ADHD typically show externalized symptoms, such as hyperactivity and impulsivity, while females tend to show internalized symptoms [25, 26]. Studies in Finland and France have also reported increased ADHD medication use among older individuals [27, 28]. For instance, in France, adults represented 34% of incident methylphenidate users. Additionally, we found that females had slightly shorter durations of ADHD medication use compared to males (see appendix II). This could be attributed to under-identification and/or absence of symptoms, and differences in coping strategies, resulting in a perceived lesser need for medication [29].

The findings of this study should be interpreted considering its limitations. Firstly, as this was an exploratory study, we conducted multiple chi-square tests without applying corrections for multiple comparisons. While this approach can increase the likelihood of Type I errors, it is appropriate for identifying potential patterns and generating hypotheses for future research. Accordingly, the findings should be viewed as preliminary and interpreted with appropriate caution.

Secondly, the database provides data from pharmacies in various regions of The Netherlands, but the exact locations of the pharmacies are not known. A study conducted in Germany observed lower ADHD medication user rates in rural regions compared to urban areas [30]. Differences in healthcare access between rural and urban regions might potentially influence dispensing patterns. However, IADB.nl database has been proven to be representative for the entire Netherlands [12].

Additionally, the Youth Act, implemented in 2015, decentralized youth mental health services from the national to municipal governments. Although it did not formally alter diagnostic or treatment thresholds, it aimed to promote integrated and preventative care. In practice, this led to variability in service organization, administrative procedures, and access to specialized care across municipalities. Implementation was neither uniform nor immediate across all municipalities, as each local government had discretion over how and when to organize services. This resulted in regional differences in timing and approach, potentially influencing observed medication rates, particularly in the years immediately following the reform. While our data, drawn primarily from the northern and eastern Netherlands, are representative, they do not allow for detailed analysis of regional disparities.

Another limitation concerns the definition for new users that was applied in this research. A new user was defined as an individual with a 90-day period without drug dispensing prior to starting a treatment. This categorization may have led to episodic ADHD medication users being classified as new users, potentially resulting in an overestimation of incidence rates. Furthermore, the duration of use may have been underestimated, and incidence rates might be overestimated due to the absence of information on dispensing during hospitalization and in-hospital medication use. Additionally, the exact age of individuals in the IADB.nl database is not known, as the database only provides dates of birth as January 1st or July 1st of the corresponding year. Consequently, when calculating ages, patients could exceed 19 years by several months. Therefore, only individuals up to the calculated age of 18.5 years were included to adhere to the age criteria of 0–19 years.

Finally, it is important to highlight that information on treatment adherence and exact indication is unavailable, and no details are available on the exact formulation of the drugs. Due to this limitation, the obtained dispensing data may not accurately reflect actual ADHD medication use with certainty. Additionally, literature reports instances of improper use of ADHD medication among students in The Netherlands [31]. This implies that the dispensations included in this study could have been distributed and not solely used for their intended treatment purposes. It is plausible that additional ADHD medication users exist but are not documented in our database. However, it is noteworthy that individuals engaging in improper use are typically not formally diagnosed with ADHD and have not obtained prescribed medication. Consequently, they would not be included in the database.

## Conclusion

The research findings indicate that the implementation of the Youth Act in 2015, which aimed to de-medicalize youth care, led to a noticeable reduction in ADHD medication dispensing. However, it is crucial to consider other influential factors that may have contributed to this trend, such as the impact of the COVID-19 pandemic, the implementation of the DSM-5, and medication shortages. Therefore, the actual impact of the Youth Act on ADHD treatment patterns is more nuanced, and the broader context of additional variables must also be acknowledged to fully understand the decline of ADHD dispensing among Dutch youth between 2010 and 2022. Further research is needed to comprehend the long-term impacts of such policy changes and to identify effective strategies for managing ADHD in youth within a decentralized healthcare system.

## Electronic supplementary material

Below is the link to the electronic supplementary material.

Supplementary Material 1

## Data Availability

The data that support the findings of this study are available in the database IADB.nl. However, these data are only made available after approval of a study protocol.
