# Supplementary material for: ADHD medication dispensing trends in Dutch youth before and after the implementation of the Youth Act (2010–2022)
Source: Eur Child Adolesc Psychiatry. 2025 Jun 23;34(12):3899–909. doi: 10.1007/s00787-025-02791-w (PMC12743109; doi:10.1007/s00787-025-02791-w)

**Table S1** Mean daily doses for the most used ADHD medication per age group in the year before implementation (2010), year of implementation (2015) and most recent year after implementation (2022).

| Drug | Age group | Mean daily dose in mg ± SD | | |
| --- | --- | --- | --- | --- |
| *Methylphenidate* | | 2010 | 2015 | 2022 |
| Males | 0-6 years | 31.3 (± 206.4) | 12.7 (± 6.6) | 13.6 (± 7.5) |
|  | 7-12 years | 23.0 (± 14.6) | 21.4 (± 21.5) | 21.5 (± 19.7) |
|  | 13-19 years | 36.7 (± 44.6) | 34.0 (± 46.9) | 33.4 (± 69.2) |
| Females | 0-6 years | 12.0 (±5.3) | 10.4 (± 6.7) | 15.9 (± 9.7) |
|  | 7-12 years | 22.7 (± 15.3) | 21.5 (± 24.6) | 20.2 (± 10.7) |
|  | 13-19 years | 31.5 (± 14.1) | 33.7 (± 63.4) | 30.9 (± 45.4) |
| *Dexamphetamine* | | | | |
| Males | 0-6 years | 11.6 (± 9.2) | 6.6 (± 4.1) | 8.2 (± 9.2) |
|  | 7-12 years | 16.9 (± 68.7) | 12.2 (± 27.0) | 10.5 (± 7.4) |
|  | 13-19 years | 28.6 (± 102.4) | 20.7 (± 94.5) | 14.2 (± 9.0) |
| Females | 0-6 years | 3.6 (± 0.5) | 4.4 (± 0.7) | 7.6 (± 6.8) |
|  | 7-12 years | 11.0 (± 4.8) | 10.7 (± 6.3) | 10.2 (± 7.6) |
|  | 13-19 years | 12.8 (± 7.7) | 15.4 (± 12.1) | 12.6 (± 7.2) |
| *Lisdexamfetamine* | | | | |
| Males | 0-6 years | Not registered | Not registered | 8.2 (± 9.2) |
|  | 7-12 years | Not registered | Not registered | 32.0 (± 11.1) |
|  | 13-19 years | Not registered | Not registered | 39.7 (± 32.6) |
| Females | 0-6 years | Not registered | Not registered | 20.0 (no SD) |
|  | 7-12 years | Not registered | Not registered | 37.3 (±16.5) |
|  | 13-19 years | Not registered | Not registered | 35.8 (± 13.5) |
| *Atomoxetine* | | | | |
| Males | 0-6 years | 24.1 (± 0.1) | 28.9 (± 14.6) | No patients |
|  | 7-12 years | 32.9 (± 12.7) | 32.1 (± 15.4) | 34.0 (± 14.6) |
|  | 13-19 years | 45.3 (± 16.7) | 67.2 (± 85.3) | 45.6 (± 25.3) |
| Females | 0-6 years | No patients | 18.2 (no SD) | 15.1 (no SD) |
|  | 7-12 years | 28.4 (± 10.1) | 31.6 (± 11.4) | 35.8 (± 16.3) |
|  | 13-19 years | 43.9 (± 16.7) | 48.2 (± 14.5) | 50.4 (± 19.6) |
| *Clonidine* | | | | |
| Males | 0-6 years | 0.3 (no SD) | No patients | 0.02 (no SD) |
|  | 7-12 years | 0.3 (± 0.1) | 0.2 (± 0.1) | 0.1 (± 0.1) |
|  | 13-19 years | 0.2 (± 0.1) | 0.2 (± 0.2) | 0.1 (± 0.1) |
| Females | 0-6 years | 0.1 (± 0.03) | 0.1 (± 0.1) | 0.02 (no SD) |
|  | 7-12 years | 0.1 (no SD) | 0.1 (no SD) | 0.1 (± 0.1) |
|  | 13-19 years | 0.3 (± 0.1) | No patients | 0.1 (± 0.1) |

Guanfacine was excluded as no patients used this drug during the above-mentioned years.

**Fig. S1 Duration of ADHD medication use between 2010 and 2022 displayed for males and females.***Each vertical line indicates a person that has stopped using ADHD medication.


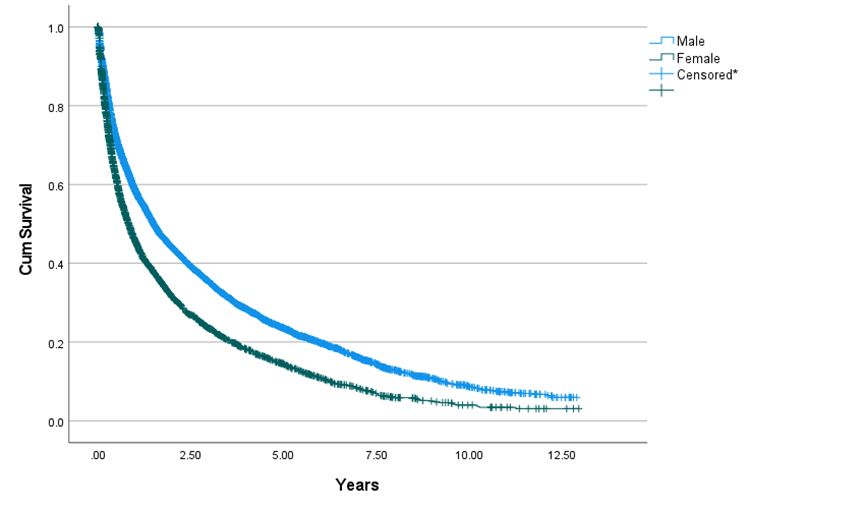

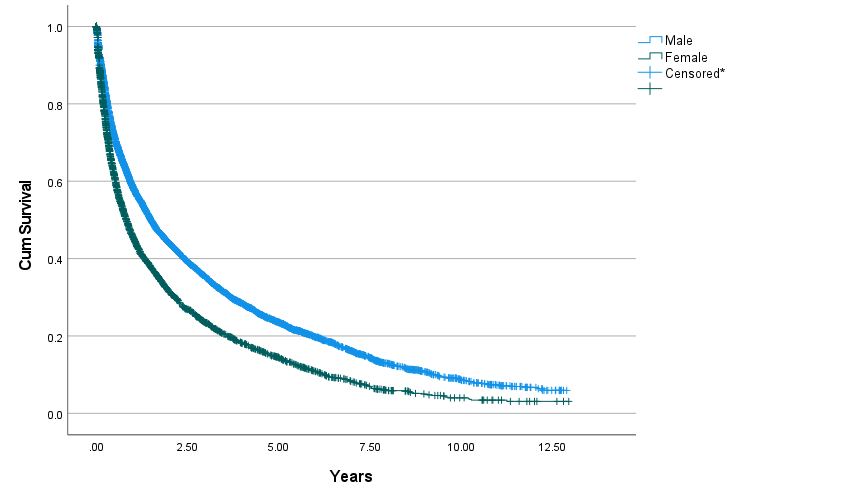

Supplement: Supplementary file 1 — Supplementary Material 1 [file 787_2025_2791_MOESM1_ESM.docx]
